# Cost-efficient strategy for reducing PM 2.5 levels in the Tokyo metropolitan area: An integrated approach with air quality and economic models

**DOI:** 10.1371/journal.pone.0207623

**Published:** 2018-11-26

**Authors:** Yushi Kunugi, Toshi H. Arimura, Kazuyuki Iwata, Eiji Komatsu, Yoshie Hirayama

**Affiliations:** 1 School of Political Science and Economics, and Research Institute for Environmental Economics and Management, Waseda University, Tokyo, Japan; 2 Faculty of Economics, Matsuyama University, Matsuyama, Japan; 3 Centre for Environmental Law and Policy, Meiji University, Tokyo, Japan; 4 Laboratory for Ecological Reconstruction Science, Tokyo, Japan; Texas A&M University, UNITED STATES

## Abstract

To attain cleaner air, it is important that authorities make informed decisions when selecting a strategy. Concentrations of particulate matter with an aerodynamic diameter of less than or equal to 2.5 μm (PM 2.5) are high in the Tokyo metropolitan area, even though concentrations of particulate matter with an aerodynamic diameter of less than or equal to 10 μm (PM10) have dropped dramatically since the implementation of the NOx-PM Act. Currently, monitored concentration levels continue to exceed the designated ambient air quality standard set by the Japanese Ministry of the Environment. To our knowledge, no study has investigated a cost-efficient strategy for reducing PM 2.5 concentration levels in the Tokyo metropolitan area. This is the first study to examine a proper control strategy for Japan by developing an integrated model that includes both aerosol and economic models. The simulation results show that prefectures in the Tokyo metropolitan area cannot achieve the standards by relying on their own efforts to reduce PM 2.5. That is, prefectural governments in the Tokyo metropolitan areas need to cooperate with prefectures outside of the area to improve their PM 2.5 concentration levels. Thus, we simulated policies under the assumption that emissions from other sources are reduced to levels such that the PM 2.5 concentration declines by approximately 18 μg/m^3^. We first simulated an efficient policy, i.e., the implementation of a pollution tax. We found that the total abatement cost to meet the air quality standard using the cost-efficient strategy is approximately 142.7 billion yen.

## Introduction

Particulate matter with an aerodynamic diameter of less than or equal to 2.5 μm (PM 2.5), an air pollutant that adversely affects human health, has attracted attention throughout the world, and particularly in China. Because of its aerodynamic, small-sized particles, which have a diameter of 2.5 micrometers or smaller, PM 2.5 causes diseases of both the respiratory and circulatory systems. According to research from the USA, if concentrations of PM 2.5 increase 10 μg/m^3^, mortality will increase 0.3%-1.2% due to short-term exposure to air pollution, or 6%-13% due to long-term exposure to air pollution [1]. Additionally, according to European research, if concentrations of PM 2.5 increase 5 μg/m^3^, mortality will increase 7% due to long-term exposure to air pollution [2]. Due to these harmful effects, high concentrations of PM 2.5 are monitored internationally [3]. The World Health Organization (WHO) [3], reported that 88% of people worldwide are exposed to PM 2.5 levels that exceed the WHO’s [4] air quality standard for annual average concentrations, which is 10 μg/m^3^. In addition to the annual average concentration, the daily average concentration is also included in the standards. Therefore, it is necessary to implement effective countermeasures. Especially we should focus on the countermeasures to achieve the air quality standard for annual average concentrations.

As in China, high PM 2.5 concentrations are also an important environmental problem in Japan. For example, in 2012, none of the roadside ambient monitoring stations in Tokyo met the Japanese ambient air quality standards for PM 2.5 [5]. One reason for this failure is that in contrast to other air pollutants, such as sulfur dioxide, nitrogen dioxide and volatile organic compounds, there is a lack of regulatory efforts targeting PM 2.5. Other pollutants have been strictly regulated by total volume control and emission standards under the Air Pollution Control Act, whereas PM 2.5 has not. Although the Japanese government has established an ambient air quality standard, it has not adopted specific regulatory measures to achieve that standard. Moreover, Japan’s standard for the annual average concentration of PM 2.5 (15 μg/m^3^), is less stringent than the WHO’s [4] standards.

To improve air quality with regard to PM 2.5 in the Tokyo metropolitan area, the regulatory authority must place much greater emphasis on stationary sources (e.g., manufacturing plants) rather than mobile sources (e.g., automobiles), even though stationary sources contribute less to total PM 2.5 emissions than do mobile sources. Around Tokyo, 36 percent and 64 percent of 2005 emissions were from stationary and mobile sources, respectively [6]. One reason is that automobiles are regulated not only by the emission standards described here but also by the stricter Top Runner Program, which regulates fuel economy [7]. Under the program, when a manufacturer introduces a new product to the market, that product’s energy efficiency must be better than that of the previous most energy-efficient product. These regulations have been gradually tightened. Another reason is that the spread of fuel-efficient hybrid and electric vehicles will reduce fuel consumption. Therefore, PM 2.5 emissions from automobiles are expected to decrease.

However, there is little evidence concerning how governments can strategically reduce PM 2.5 emissions from stationary sources around Tokyo. To the best of our knowledge, there is no research focusing specifically on the strategic reduction of PM 2.5 in Japan. This shortage of evidence contrasts remarkably with the situation in Europe, where many studies of PM 2.5-reduction strategies have been conducted [8–11]. Therefore, using simulation analysis with several scenarios, this paper aims to examine an ex-ante cost-efficient strategy for reducing PM 2.5 emissions from stationary sources in the Tokyo metropolitan area; we take this approach because cost-efficiency is one of the most universal criteria for policymaking [12]. That is, we will propose a strategy that can meet the area’s air quality standards at the lowest abatement cost. Using 56 control measures provided by the Environmental Protection Agency (EPA) [13], we find an optimal combination of implementing control measures at minimum cost in order to achieve the ambient standard. To our knowledge, this is the first study to examine a proper strategy for Japan. Several studies have conducted ex-post evaluations on air pollution regulations in Japan, for example, evaluations automobile regulations against exhaust gas emissions [14, 15].

It is quite helpful, for our purposes, to survey the European situation. Unlike Japan, Europe has built excellent monitoring databases and integrated modeling systems for simulation of PM 2.5. A key feature of the European approach is the use of an integrated modeling system such as the Regional Air Pollution Information and Simulation (RAINS) model [8, 16, 17], which includes both aerosol and abatement cost models. There are also other models targeting the UK [18, 19], or The Greenhouse Gas and Air Pollution Interactions and Synergies (GAINS) model [9–11, 20–23], which take into consideration greenhouse gases in recent years. In addition, GAINS is used not only in Europe but also in Asian countries such as China and India where air pollution problems are serious [24–26] or occur in more global air [27]. However Japan has not yet used these models. These fundamental components enable us to examine the strategy for controlling PM 2.5 emissions as well as sulfur dioxide and nitrogen dioxide emissions [28–30]. In Japan, monitoring databases such as the Atmospheric Environmental Regional Observation System (AEROS) [31, 32] and the Japan Auto-Oil Program (JATOP) [33], as well as numerical air quality models such as the Spectral Radiation-Transport Model for Aerosol Species (SPRINTERS) [34, 35] and Atmospheric Dispersion Model for Exposure and Risk Assessment PRO (ADMER-PRO) [36–39], are available; however, abatement cost models are not. Therefore, abatement cost models must be developed and incorporated into the integrated modeling systems, as has been done in RAINS and GAINS.

The features of our research are as follows. First, in our research, both abatement costs and air quality models are integrated in order to achieve our goal. Of course, many previous studies have also used integrated simulation models [8–11, 16–23]. However, in these studies, they consider the cost minimization to achieve goals already decided upon for each region and atmospheric conditions at that time. Europe and America accede to the Convention on Long-Range Transboundary Air Pollution, and they have reduction targets of some air pollutants for each region under this convention or under other policies. On the other hand, Japan has no concrete reduction target. Therefore, we first need to decide upon reduction targets of PM 2.5 when we analyze our model. However, this is a convenient approach because we can consider reduction targets that minimize reduction costs, taking into account the contribution of pollutants between regions.

Second, this paper addresses both primary and secondary PM 2.5 emissions using JATOP [33]. The primary emissions are generated from fuel combustion at plants. Secondary emissions result from chemical reactions in the atmosphere; thus, we account for particle conversions of atmospheric and other material compounds into PM 2.5. Secondary PM 2.5 compounds should not be neglected, as approximately 60% of total emissions are attributable to them [40]. We select sulfur and nitrogen compounds (SOx and NOx) for analysis using abatement cost models, but we omit ammonia and volatile organic compounds because they have no available inventory regarding abatement cost. However, when we perform analyses using air quality models, we consider the influence of ammonia and volatile organic in atmospheric chemical reaction processes.

Third, we use ADMER-PRO [36] as the air quality modeling system. This model was developed as a successor model of ADMER (National Institute of Advanced Industrial Science and Technology-Atmospheric Dispersion Model for Exposure and Risk Assessment: AIST-ADMER) [41–43] by the National Institute of Advanced Industrial Science and Technology, Japan. We decided to use this model because it is fitting for the analysis of atmospheric chemical reactions within a medium scale area such as the Tokyo metropolitan area. ADMER-PRO [36] is a Eulerian chemical transport model coupled with a meteorological model. The meteorological model is applied to the Regional Atmospheric Modeling System (RAMS) [44] and can be used to estimate the spatial dispersion of air pollutants from one area to others. The gas-phase photochemistry mechanism in this model is applied to the Carbon Bond Mechanism IV (CBM-IV) [45] for a better treatment of chemistry in regional environments. Using this model, we can calculate particle transformation of atmospheric and other material compounds into PM 2.5.

Fourth, our abatement cost model uses control measures that are consistent with reality, as all measures are not always available in all industries. For example, a fabric filter can be installed at plants in the steel industry but cannot be used in the chemical industry. We refer to [46] as our list of available measures; this list reports the abatement costs and elimination performances of various measures. Although the values are for the United States, we assume no differences between the United States and Japan with regard to the costs and performances because no relevant information is available for Japan.

Fifth, using the JATOP [33] database, we include almost all stationary sources and their primary PM 2.5, SOx and NOx emissions in Tokyo’s metropolitan areas. The coverage rate is very high, at 95.8% of total emissions. In the database, the Tokyo metropolitan area is divided into 38,300 one-kilometer mesh grids, where emissions of each pollutant from each fuel type in each industry are reported. A total of 11 industries and 38 fuel types are included (see S1 Table and S2 Table) because of the described technological constraints on measurements and the different conversion rates for the three pollutants among fuel types. This detailed dataset enables us to precisely simulate PM 2.5 concentration levels in the Tokyo metropolitan area.

**Fig 1:**
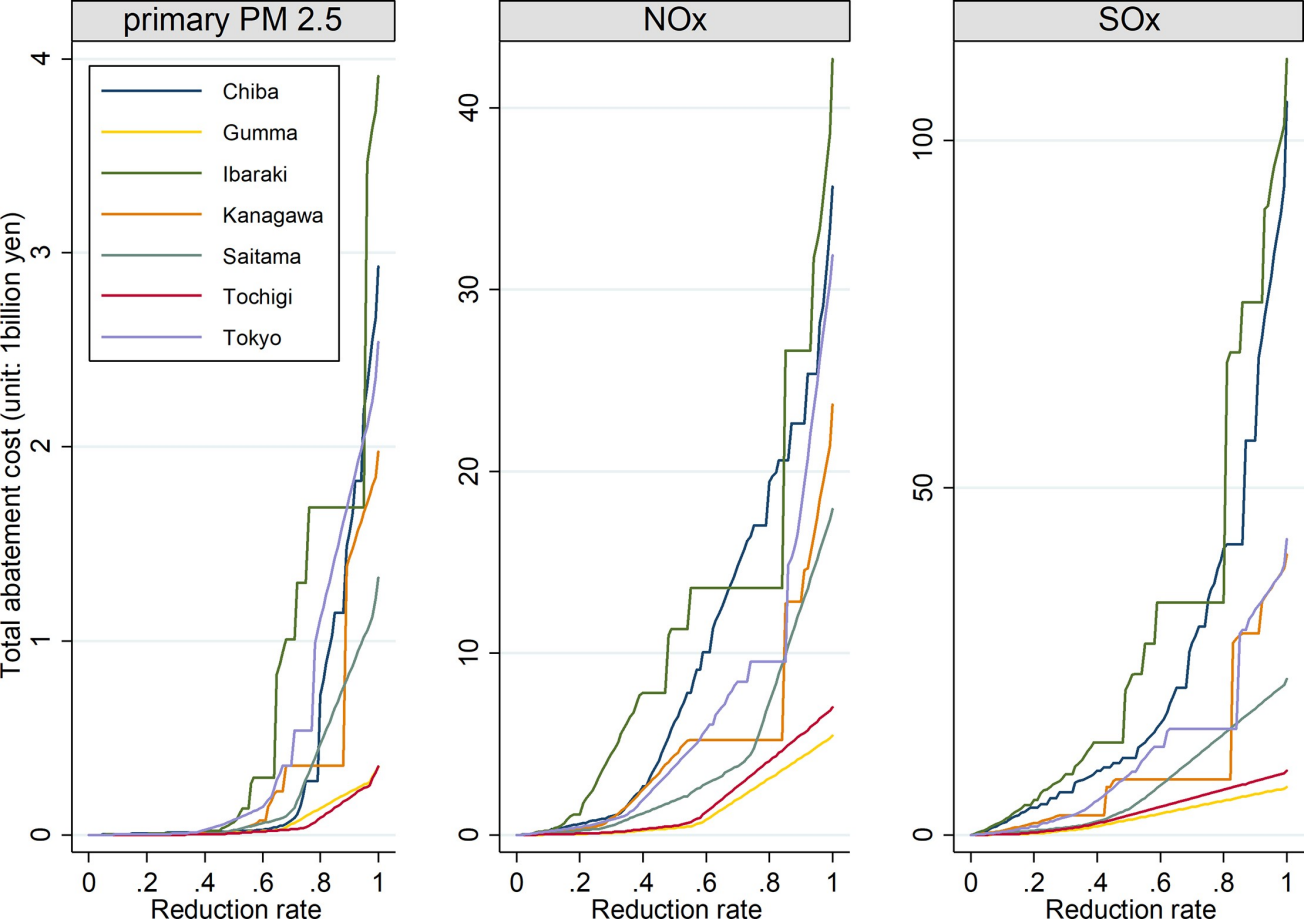
Seven prefectures in the Tokyo metropolitan area.

Sixth, aggregating 38,300 one-kilometer mesh grids into 7 prefectures (i.e., Tokyo, Kanagawa, Chiba, Saitama, Ibaraki, Tochigi and Gunma prefectures, as illustrated in Fig 1), a spatial dispersion of pollutants among the prefectures is included in our air quality model, implying that emissions reductions achieved through the implementation of some measures in one prefecture contribute to the reduction of concentration levels of emissions in the other prefectures via dispersion. Suppose that one prefecture mandates that facilities located therein must install PM 2.5 control measures. Concentration levels in neighboring prefectures also decline due to the installation, even if those prefectures took no action to reduce PM 2.5. In this case, the neighboring prefectures may not implement any regulations against PM 2.5. This is known as a free-rider problem [47], which leads to improvements in air quality, but at higher abatement costs than what would be optimal/minimal. To avoid this problem, this paper presents well-constructed individual blueprints for each prefecture, highlighting which control measures should be adopted in each area.

We can summarize our results as follows: First, even if all prefectures in the Tokyo metropolitan area were to mandatorily install all available control measures, the predicted PM 2.5 concentration levels would still exceed air quality standards, implying that meeting the standards requires external countermeasures such as mobile source controls and the implementation of measures outside the Tokyo metropolitan area. Second, even if we assume the presence of exogenous external countermeasures, our simulation results show large differences in total abatement costs among strategies, which will be explained in Section 2. In certain scenarios, the cost gap is enormous (up to 44 times). This suggests that a sophisticated analysis is necessary to plan a rational strategy to combat PM 2.5. Moreover, the results confirm the importance of economic instruments, such as a pollution tax, to lower the cost of compliance [12].

The remainder of this paper is organized as follows: In Section 2, we explain our integrated simulation model. Section 3 presents the dataset used in this paper. Section 4 presents our simulation results and the policy implications derived from those results. Section 5 concludes the paper.

## Integrated simulation models

### Abatement cost model

Our simulation model consists of two parts: the abatement cost model and the air quality model (spatial emission concentration model). In this section, we explain the abatement cost model and then present the spatial emission concentration model. We express the abatement costs of a control measure for pollutant *p*(*p* = 1,2,3) from fuel type *f*(*f* = 1,…,38) at *m*^*i*^th mesh ($m^{i}=1^{i},\ldots ,M^{i}\forall \sum_{i=1}^{7}M^{i}=38,300$) in prefecture *i* (*i* = 1,…,7) as $C_{m^{i}f}^{p}$. We also address the abatement cost by industry (*j* = 1,…,11), and the cost can be written as $C_{m^{i}fj}^{p}$. The maximum number of combinations (i.e., individual sources potentially emitting primary PM 2.5, SOx or NOx) is 48,028,200 (= 3×38×11×38,300). However, all fuel types are not used in all industries, and all industries are not located in all meshes. This suggests that we cannot cut emissions from all individual sources. Therefore, after removing the sources for which reduction potentials are zero, the number of individual sources eventually drops to 649,386, where some control measures can be applied. It is assumed that multiple identical control measures cannot be introduced at one individual source. Additionally, we assume that the control measure for one pollutant from one fuel type in one industry is of only one type. Further, we introduce an assumption that any control measures listed in the EPA’s inventory [46] have not yet been installed in the Tokyo metropolitan area. The total abatement cost in prefecture *i*, therefore, is described as

$$C_{i}=\sum_{m^{i}}\sum_{f}\sum_{j}\sum_{p}C_{m^{i}fj}^{p}\times D_{m^{i}fj}^{p}$$ (1)

where $D_{m^{i}fj}^{p}$ is an indicator variable that takes a value of 1 if a control measure is installed and otherwise takes a value of 0. The total abatement cost in the Tokyo metropolitan area (*TC*) is expressed as the sum of *C*_*i*_ on prefectures, that is, *TC* = ∑_*i*_*C*_*i*_.

### Spatial emission concentration model

First, let the emission reduction of pollutant *p* at the individual source be ${ER}_{m^{i}fj}^{p}$. The pollutant removal rates are expressed as $r_{m^{i}fj}^{p}$, which take a value between 0 and 1. When the measure perfectly removes the pollutant emissions, the variable equals a value of 1. The emission reduction performances of control measures are technologically predetermined. Then, using the indicator variable, the total emission reduction of pollutant *p* in prefecture *i* is described as follows:

$${ER}_{i}^{p}=\sum_{m^{i}}\sum_{f}\sum_{j}{\bar{E}}_{m^{i}fj}^{p}\times r_{m^{i}fj}^{p}\times D_{m^{i}fj}^{p}$$ (2)

The overall reduction of the pollutant *p* in the Tokyo metropolitan area is expressed as ${TER}^{p}=\sum_{i}{ER}_{i}^{p}$. The emissions of pollutant *p* without any installation of control measures (i.e., emissions in the business-as-usual (BAU) scenario) are provided by the JATOP [33] database. ${\bar{E}}_{m^{i}fj}^{p}$ denotes the emissions. Then, the total emissions after the installation in prefecture *i*, $E_{i}^{p}$, are written as Eq (3).

$$E_{i}^{p}=\sum_{m^{i}}\sum_{f}\sum_{j}\left({\bar{E}}_{m^{i}fj}^{p}-{\bar{E}}_{m^{i}fj}^{p}\times r_{m^{i}fj}^{p}\times D_{m^{i}fj}^{p}\right)$$ (3)

Next, the emissions of three pollutants ($E_{i}^{p}$) are converted into annual average PM 2.5 concentration levels by introducing spatial dispersions and particle conversions. $a_{oi}^{p}$ is the contribution rate of the emissions of pollutant *p* in prefecture *o* to the PM 2.5 concentration level in prefecture *i*. The prefecture *i*’s own contribution rate of the pollutant *p*, $a_{ii}^{p}$, indicates that the PM 2.5 concentration level is affected by the remainder of the emissions of pollutant *p*. Using the contribution rates, the PM 2.5 concentration level in prefecture *i* (*AQ*_*i*_) is described as follows:

$${AQ}_{i}=\sum_{p=1}^{3}\sum_{o=1}^{7}a_{oi}^{p}E_{o}^{p}$$ (4)

Because PM 2.5 remains in the atmosphere for approximately 10 days [48], we assume that the emissions of each pollutant at year *t* influence the PM 2.5 concentration level only at year *t*, implying that dynamic factors such as discount rate and economic growth are not included in our static simulation model. Replacing $E_{o}^{p}$ with ${\bar{E}}_{o}^{p}$ in Eq (4), we can obtain the PM 2.5 concentration level in the BAU scenario, ${\bar{AQ}}_{i}$. Therefore, the concentration reduction is written as ${\bar{AQ}}_{i}-{AQ}_{i}$. Recall that our focus is on the installation of control measures on stationary sources. Therefore, the total concentration level is the sum of *AQ*_*i*_ and the concentrations from other sources (i.e., natural events, mobile sources and emissions outside the Tokyo metropolitan area). The concentration caused by the other sources is described as $\tilde{X}.\;\tilde{S}$ represents the air quality standard designated by the Japanese EPA. Then, the total concentration levels in all areas must be less than $\tilde{S}$, as in Eq (5). Our proposed strategies are required to satisfy the following constraint:

$${AQ}_{i}+\tilde{X}\leq \tilde{S}\;for\;all\;i$$ (5)

Although the contribution rates are set between any two areas (i.e., *a*_*oi*_), ideally, they should be set between any two one-kilometer mesh grids (i.e., $a_{m^{o}m^{i}}$). However, for computational feasibility, we employ them at the prefecture level. We denote the PM 2.5 concentration level in prefecture *i*, completely excluding the effect of pollutant *p* from prefecture *o*, as *AQ*_*i*,*op* = 0_. Then, contribution rates of *p* from *o* to *i* can be calculated as $a_{oi}=\left({\bar{AQ}}_{i}-{AQ}_{i,op=0}\right)/{\bar{AQ}}_{i}$. For calculating contribution rates and PM 2.5 concentrations under various scenarios, we use ADMER-PRO [36] which estimates the atmospheric concentrations of chemical substances.

### Optimization problems

The total concentration levels decrease with increasing installations of control measures, while the increase in installations raises costs. Therefore, the best cost-efficient strategy is one that minimizes total abatement cost (Eq (1)) and that satisfies the air quality standard in each prefecture (Eq (5)). The optimization problem for the strategy is expressed as follows:

$$\begin{array}{c} \min_{D_{m^{i}fj}^{p}}TC\\ s.t.\;{AQ}_{i}+\tilde{X}\leq \tilde{S}\;for\;all\;i \end{array}$$ (6)

Solving this problem, we can find the optimal combination of ${\hat{D}}_{m^{i}fj}^{p}$, which is the first best solution.

However, such a solution cannot be practically introduced if people favor a strategy that is equal among prefectures, as equality often deteriorates as cost-efficiency improves [49]. The most cost effective strategy may impose disproportionately large costs on a specific region or prefecture. Therefore, in addition to the cost-efficient strategy, we consider another optimization problem as a second-best strategy. That is, we pay attention to equality among prefectures when abatement technologies are installed. Equality is defined as the imposition of uniform reduction rates on all prefectures; these rates are emissions reductions divided by total reduction potentials (note that the reduction rates are not reduction amounts per type of emissions). The average reduction rates for each prefecture are described as ${\dot{r}}_{i}^{p}$. Adding the new constraint of equality to Eq (6), we rewrite the optimization problem for the second-best strategy as the following, Eq (7).

$$\begin{array}{c} \min_{D_{m^{i}fj}^{p}}TC\\ s.t.\;\begin{array}{c} {AQ}_{i}+\tilde{X}\leq \tilde{S}\;for\;all\;i\\ {\dot{r}}_{i}^{p}={\dot{r}}_{o}^{p}\forall i,o,where\;i\neq o \end{array} \end{array}$$ (7)

We denote the cost-efficient strategy and the uniform rate strategy as the CES and URS scenarios, respectively. In both scenarios, it is assumed that the prefectures install the control measures in order to achieve low abatement costs per emission reduction (i.e., marginal abatement costs).

## Data description

The primary PM 2.5, SOx and NOx emissions from stationary sources in 2005 are obtained from the JATOP [33] database which is estimated by the Japan Petroleum Energy Center (JPEC) [50–53]. Anyone can apply for using the database via email inquiry (JPEC [50]). The database covers approximately 95.8% of total emissions and provides these emissions by spatial one-kilometer mesh, by fuel type and by industry. Table 1 shows the total emissions for each pollutant and the total number of individual sources by prefecture. The total number of individual sources is 649,386. Of these, 41,236 sources emitting primary PM 2.5 are located in Tokyo. We find that there are more sources emitting primary PM 2.5 than there are sources emitting other pollutants. Viewing emissions volumes by prefecture, every pollutant is emitted more in Chiba and Ibaraki, because these prefectures have many sources of high emissions, such as coal-fired power plants, steel plants and oil refinery plants. In contrast, lower emissions are found in Gunma and Tochigi, where, due to the inland location, no plants with high emissions have been constructed (see Fig 1). Although NOx and SOx emissions are not converted into the ton of PM 2.5 equivalent in Table 1, the total volume of primary PM 2.5 emissions is much smaller than the total volumes of the other two pollutants. From the introduction of environmental standards in Japan (2007) to the present (2015), the atmospheric concentration of PM 2.5 has been decreasing [54] in the Tokyo metropolitan area. The situation embodied by our data (2005) seems to be far from the present situation. However, the current decrease in PM 2.5 is not necessarily due to specific measures such as cost-benefit strategies that takes cost minimization into consideration. Therefore, using data before the introduction of environmental standards will be important to compare our study with the future ex-post evaluation of the reduction in Japan.

We obtain the list of control measures, their annual average abatement costs ($C_{fj}^{p}$) and their emissions reduction performances ($r_{fj}^{p}$) from the Control Strategy Tool (CoST) [55] provided by the EPA [44]. According to the CoST [55], for example, the installation of a dry electrostatic precipitator (wire plate type) in the black liquor recovery process (in the wood pulp and paper product industry) can eliminate 95% of primary PM 2.5 emissions, and its average cost is 11,000 yen per year (approximately 100 yen are equal to 1 US dollar) per ton of PM 2.5. As another example, in the mineral products industry, a fabric filter (pulse jet type) can remove 99% of primary PM 2.5 emissions at a cost of 11,700 yen per year per ton of PM 2.5. These examples show that both reduction performances and costs can differ among control measures. Using the JTOP database [33] and information from the EPA [46], we can calculate the detailed reduction performances and costs, depending on control measures by the fuel types of each industry. At this time, we assume that the ratio of fuel types used in each industry does not change before and after introducing control measures. Therefore, if some industries change the ratio of fuel types after introduction control measures, we should to be aware that there may be some uncertainty in our estimate.

Using the procedure of the Environmental Restoration and Conservation Agency (ERCA) [56], we match individual sources with the list of measures. Table 1 also presents the reduction potentials and reduction rates by prefecture, when all available control measures are implemented. We express this case as the Extreme (EX) scenario. The total reduction potentials of primary PM 2.5 are smaller than the potentials of NOx and SOx. The reduction potential rates are calculated as reduction potentials divided by emissions. In the EX scenario, the bold implementation can cut 93% of total SOx emissions, whereas only 65% of total primary PM 2.5 emissions can be removed. This implies that it is technologically more difficult to purge primary PM 2.5 emissions compared with the other two types of emissions. The rightmost column in Table 1 shows the total abatement costs for each pollutant and each prefecture in the EX scenario. The total abatement cost of primary PM 2.5 is 13.4 billion yen per year, while the installations for SOx and NOx cost 164.2 and 339.1 billion yen, respectively. These figures suggest that large (small) amounts of SOx (primary PM 2.5) emissions can be cut at higher (lower) cost. As mentioned in Section 2, our simulation model is static. Therefore, the term, “per year,” is set aside.

**Table 1:** Emissions, reduction potentials and abatement costs by pollutant and by prefecture.

primary PM 2.5					
Prefecture	Number of individual sources in 2005	Emissions in 2005 (ton)	Reduction potentials (ton/year)	Reduction potential rates (%)	Total abatement costs (1 billion yen/year/ton)
Chiba	55,283	3,389	2,262	67%	2.9
Gunma	39,914	246	155	63%	0.3
Ibaraki	81,736	2,549	1,643	64%	3.9
Kanagawa	26,966	1,263	878	70%	2.0
Saitama	64,265	1,040	684	66%	1.3
Tochigi	46,867	295	196	66%	0.4
Tokyo	41,236	1,955	1,169	60%	2.5
Total	356,267	10,737	6,987	65%	13.4
NOx (secondary PM 2.5)					
Prefecture	Number of individual sources in 2005	Emissions in 2005 (ton)	Reduction potentials (ton/year)	Reduction potential rates (%)	Total abatement costs (1 billion yen/year/ton)
Chiba	22,744	53,187	41,363	78%	35.6
Gunma	14,961	3,204	2,088	65%	5.5
Ibaraki	31,321	45,637	32,998	72%	42.7
Kanagawa	11,318	24,965	18,513	74%	23.7
Saitama	27,192	13,743	9,685	70%	17.9
Tochigi	17,380	4,025	2,617	65%	7.0
Tokyo	17,020	35,368	25,556	72%	31.9
Total	141,936	180,129	132,820	74%	164.2
SOx (secondary PM 2.5)					
Prefecture	Number of individual sources in 2005	Emissions in 2005 (ton)	Reduction potentials (ton/year)	Reduction potential rates (%)	Total abatement costs (1 billion yen/year/ton)
Chiba	24,191	40,565	38,257	94%	105.7
Gunma	15,447	2,646	2,353	89%	7.0
Ibaraki	31,657	54,819	51,577	94%	111.8
Kanagawa	13,841	18,955	17,702	93%	40.4
Saitama	29,991	10,392	9,478	91%	22.5
Tochigi	16,326	3,542	3,158	89%	9.2
Tokyo	19,730	17,931	16,635	93%	42.6
Total	151,183	148,850	139,161	93%	339.1

**Fig 2:**
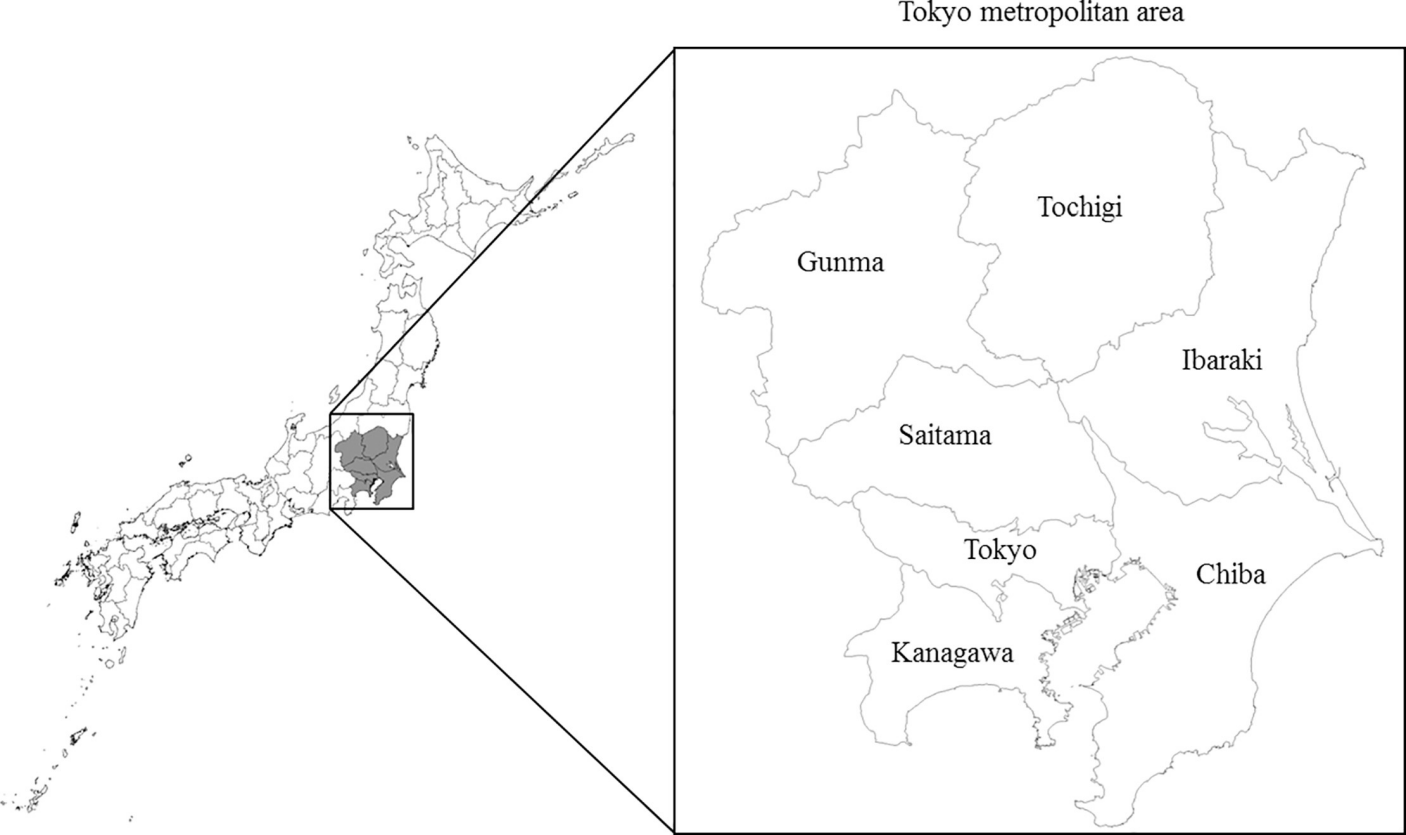
Relationship between total abatement costs and reduction rates by pollutant and by prefecture.

Fig 2 shows the relationship between the total abatement costs and reduction rates by prefecture and by pollutant. In the figure, the horizontal axis represents reduction rates rather than emission reductions. As the reduction rates increase, the total abatement costs also increase in all prefectures. We find some consistent features across all relationships. This is because one control measure can generate significant emission reductions at one individual source, such as a coal-fired power plant. The shapes of the relationships are found to be different among prefectures. For each reduction rate, Gunma always spends the least on reducing SOx, whereas the total cost spent on SOx in Ibaraki is always highest, except when the reduction rate is approximately 0.77. This implies that to achieve a cost-efficient strategy, we must find a careful balance between the abatement cost and emissions reductions.

## Simulation analyses

### Results under the extreme scenario

Table 2 presents the simulation results under the EX scenario. The second column, titled “2005,” represents the observed annual average PM 2.5 concentration levels by prefecture in 2005. The concentration levels in all prefectures exceed the air quality standard for annual average concentrations. The concentration levels in the BAU scenario are presented in the third column. As mentioned earlier, PM 2.5 emissions from automobiles are expected to decline, a reduction that is taken into account in the BAU scenario. Therefore, the concentration levels decrease by 1.0 to 2.1 μg/m^3^ in the BAU scenario. Regarding the reductions attributed to automobiles, we refer to the estimation in JATOP [33].

The column titled “EX scenario” represents the concentration level in the EX scenario. In this scenario, the reduction rates are fixed at 1 in all prefectures. Comparing the concentration levels with the air quality standard, we find that Ibaraki and Tochigi prefectures satisfy the standards. In contrast, the other five prefectures cannot meet the standards if they rely only on their efforts to reduce the three pollutants from stationary sources. This result leads to an important policy implication: If emissions from automobiles and natural events cannot be reduced by control policies, the prefectures need to call for cooperation with the other prefectures outside the Tokyo metropolitan area (or overseas countries) in order to improve air pollutant concentrations around Tokyo [57].

**Table 2:** Comparison of concentration levels (under the EX scenario) with the air quality standard.

	PM 2.5 concentration level			Air Quality Standard
Prefecture	2005	BAU scenario	EX scenario	
Chiba	20.9	19.9	17.5	15
Gunma	19.3	17.9	15.8	15
Ibaraki	17.9	16.5	13.6	15
Kanagawa	22.1	20.7	18.3	15
Saitama	23.4	21.3	17.5	15
Tochigi	19.1	17.2	14.4	15
Tokyo	21.3	19.7	16.8	15

All units are μg/m^3^.

### Results under the cost-efficient strategy and uniform reduction strategy scenarios

Even when all control measures are installed, not all of the prefectures can meet the air quality standards, implying that we cannot solve the optimization problems as presented in Eqs (6) and (7). If the concentration levels from outside the metropolitan area were to decrease by 3.5 μg/m^3^, all prefectures would be able to achieve clean air, thus satisfying the standard. Therefore, for the simulation analysis under the URS and CES scenarios, we assume that the external concentration reductions of 3.5 μg/m^3^ (i.e., $\tilde{X}=3.5$) are generated in all prefectures. This value is the valid result of calculation. First, we placed the area outside of the Tokyo metropolitan area as a single area. We then estimated the concentration of outside contributions to the Tokyo metropolitan area using ADMER-PRO [36]. In addition, we estimated the emission reduction and the abatement cost of the outside area with outside emission data and a reference to our estimated cost data of the Tokyo metropolitan area. As a result, we determined that 3.5 μg/m^3^ is an efficient value for lowering the concentration in Tokyo. Therefore, we decided to use 3.5 μg/m^3^ as the external reduction value.

Based on the external concentration reductions of 3.5 μg/m^3^, Table 3 shows the estimated results of reduction rates and emissions reductions under the CES and URS scenarios. Our integrated model consists of the nonsmooth abatement cost (Eq (1)) and emission concentration functions (Eq (4)) because the indicator variable ($D_{m^{i}fj}^{p}$) is discrete. These characteristics prevent us from finding a solution for the variable using a nonlinear programming method. Therefore, in order to accelerate the calculation time, after we approximate the relationship between abatement cost and emission concentration to exponential function form, a tentative solution is obtained by a nonlinear programming method. Going back to the original functions and setting the obtained tentative solution to an initial value, we search for a correct solution in each neighborhood by the grid search method. Under both scenarios, the PM 2.5 concentration levels are equal to the air quality standards in every prefecture. The cost-efficient strategy requires authorities to designate the estimated reduction rates under the CES scenario. The rates are found to vary across prefectures and across pollutants, implying that tailored designations are necessary to achieve cost-efficiency. Under the CES scenario, the reduction rates of primary PM 2.5 and NOx in Kanagawa are 100%, suggesting that all measures used to control those pollutants should be installed in Kanagawa. In contrast, in Chiba, the rates of primary PM 2.5 and NOx are estimated to be 85% and 55%, respectively. The spatial dispersion of pollutants generates the differences in reduction rates among prefectures. Generally, the air flow takes the form of westerlies heading from east to west over Japan [58]. Therefore, we find low reduction rates in Chiba and Ibaraki, which are located east of the Tokyo metropolitan area (see Fig 1). Among pollutants, the reduction rates of SOx are lower than those of the other pollutants. The gap results from the differences in particle conversions and abatement costs. This result implies that the reduction measures for SOx cost more than the reduction measures for primary PM 2.5 and NOx. Therefore, authorities in the Tokyo metropolitan area should focus their efforts on pollutants in this order: primary PM 2.5, NOx and SOx. In the URS scenario, the uniform reduction rates are 95%. If it were to adopt the URS scenario, Kanagawa would introduce fewer control measures compared to the case of the CES scenario. In contrast, more installations are required to meet the air quality standards in the other prefectures.

Table 4 presents the total abatement costs by prefecture and by pollutant under the two scenarios. The remarkable point is the difference in total abatement costs between the two scenarios: 142.7 billion yen and 416.3 billion yen under the CES and URS scenarios, respectively. The latter is approximately 2.9 times greater than the former, implying that uniform regulation, relative to cost-efficient regulation, generates significant costs without consideration of each prefecture’s situation. Consistent with economic theory, our paper also finds a tradeoff between equality and cost-efficiency [49]. Furthermore, when equality in reduction rates is maintained under the URS scenario, the equality of cost burdens deteriorates. In the CES scenario, the total abatement costs in Ibaraki and Gunma are 8.4 and 28.6 billion yen, respectively, with the latter being approximately 3.4 times greater than the former. In contrast, the difference between the two prefectures is approximately 11.3 times in the URS scenario (11.4 and 128.5 billion yen in Gunma and Ibaraki, respectively). As a robustness check, we conduct four additional simulations where the external concentration reductions are assumed to be 5.0 μg/m^3^ (see S3 Table and S4 Table). Regardless of the extent of the external concentration reduction, our findings are consistent.

**Table 3:** Reduction rates and emissions reductions by prefecture under the CES and URS scenarios.

Reduction Rates (%)						
	CES scenario			URS scenario		
Prefecture	primary PM 2.5	NOx	SOx	primary PM 2.5	NOx	SOx
Chiba	85%	55%	55%	95%	95%	95%
Gumma	95%	85%	75%	95%	95%	95%
Ibaraki	95%	84%	48%	95%	95%	95%
Kanagawa	100%	100%	82%	95%	95%	95%
Saitama	90%	75%	65%	95%	95%	95%
Tochigi	90%	75%	75%	95%	95%	95%
Tokyo	95%	73%	84%	95%	95%	95%
Emissions Reductions (ton)						
Chiba	1,922	22,206	20,942	2,149	38,036	36,343
Gumma	146	1,774	1,764	146	1,983	2,235
Ibaraki	1,244	17,856	20,046	1,244	31,345	48,997
Kanagawa	878	18,513	8,034	834	17,587	16,817
Saitama	615	7,262	6,158	649	9,199	9,003
Tochigi	176	1,962	2,367	185	2,486	2,999
Tokyo	1,109	17,794	10,431	1,109	24,270	15,793
Total	6,090	87,367	69,742	6,316	124,906	132,187

**Table 4:** Total abatement cost by prefecture under the CES and URS scenarios.

	CES scenario				URS scenario				Difference
Prefecture	primary PM 2.5	NOx	SOx	Total (A)	primary PM 2.5	NOx	SOx	Total (B)	(B)/(A)
Chiba	1.1	7.8	13.3	22.2	2.2	25.3	80.6	108.1	4.9
Gunma	0.3	3.7	4.5	8.4	0.3	4.8	6.3	11.4	1.4
Ibaraki	1.7	13.6	13.3	28.6	1.7	32.6	94.2	128.5	4.5
Kanagawa	2.0	23.7	8.0	33.6	1.6	17.4	35.6	54.6	1.6
Saitama	0.8	4.8	9.0	14.6	1.0	15.1	20.2	36.3	2.5
Tochigi	0.2	3.4	5.9	9.5	0.2	6.2	8.4	14.9	1.6
Tokyo	2.0	8.4	15.3	25.8	2.0	24.8	35.6	62.4	2.4
Total	8.1	65.2	69.3	142.7	9.1	126.3	280.9	416.3	2.9

All units are 1 billion yen.

## Discussion

We propose a third avenue for achieving cost-efficiency. It may be difficult for prefectures to implement tailored installations of control measures on each individual source (as shown in Table 3) because the administration costs may be high. To overcome this problem, economic theory suggests an environmental tax (i.e., tax per ton of pollutant) [12]. An emission trading scheme is also considered a cost-efficient instrument, similar to an environmental tax. Unlike a command-and-control approach such as the tailored designations, under the environmental tax scenario, prefectures only need to impose a uniform tax rate on all individual sources. Balancing installation costs and tax burdens, the individual sources then decide whether to install control measures. Consequently, cost-efficiency is attained when the marginal abatement costs are equal to the uniform tax rate. An important point is that the decisions depend on the individual sources. Therefore, in implementing the tax, the prefectures do not need to carefully examine the availabilities of the control measures for each source.

However, an environmental tax with a uniform tax rate cannot be a first-best instrument in a situation wherein polluters and receptors are in different locations [12], which is precisely the situation described in our paper (i.e., the large-scale dispersion of pollutants). This is because the marginal damages of pollutant emissions are different depending on the locations of the polluters and receptors. In this paper, damage is interpreted as contributions to the increase in PM 2.5 concentration level. As mentioned above, we introduce the exogenous parameter *a*_*oi*_ to capture the spatiality; that is, the effects of the polluters in prefecture *o* on the receptors in prefecture *i*. Using this parameter, the uniform tax rate must be adjusted among prefectures and pollutants (For detailed arguments, see [12].).

Consistent with the economic theory described above and given the external concentration reductions of 3.5 μg/m^3^, in order to satisfy the standards in each prefecture, the cost-efficient tax rates must be equal to the marginal abatement costs under the CES scenario shown in Table 5. The marginal abatement costs are calculated as the total abatement cost (shown in Table 4) divided by the emissions reductions (shown in Table 3). If Kanagawa intends to introduce an environmental tax on PM 2.5, they must set the tax at 2.3 million yen per ton of primary PM 2.5. In contrast, the tax rate for primary PM 2.5 is lowest in Chiba, at 0.6 yen million yen per ton. The difference is due to the locations of the two prefectures (see Fig 1). Even in Chiba, the tax rates are different among pollutants: 0.6, 0.4 and 0.6 million yen per ton of primary PM 2.5, NOx and PM, respectively. The difference within the same prefecture is due to the difference in the particle conversion rates. When each prefecture introduces tax rates for each pollutant, the total abatement costs are theoretically consistent with those of the tailored designations under the CES scenario, and eventually they achieve clean air with concentration levels that are just equal to the air quality standard. In addition, unlike under the designations, tax revenues are generated for the prefectures.

**Table 5:** Marginal abatement cost by prefecture and by pollutant.

Prefecture	primary PM 2.5	NOx	SOx
Chiba	0.6	0.4	0.6
Gumma	1.8	2.1	2.5
Ibaraki	1.4	0.8	0.7
Kanagawa	2.3	1.3	1.0
Saitama	1.4	0.7	1.5
Tochigi	1.1	1.7	2.5
Tokyo	1.9	0.5	1.5

## Concluding remarks

Many researchers are seriously concerned about PM 2.5 concentrations in Japan because PM 2.5 is more harmful to human health than conventional PM 10, which is already regulated by several policies. In 2012, none of the roadside ambient monitoring stations in Tokyo achieved the stated air quality standards [5]. Despite the harmful effects and high concentration levels of PM 2.5, effective policies and strategies to reduce this pollutant have not yet been planned or implemented. One reason is that unlike Europe, where integrated models such as the RAINS [8] or GAINS [9] have been developed, Japan has no integrated model that can be used to examine effective control strategies. Integrated models such as the RAINS [8] or GAINS [9] are required to consist of two models, an air quality model and economic model. If we want to identify an appropriate strategy, we can ignore neither abatement costs nor improvements in air quality. Although many researchers in Japan have focused on improving the air quality model, there has been little discussion of the economic model.

Therefore, by integrating the air quality model and a new economic cost model into a single model, the purpose of this paper is to examine a cost-efficient strategy for reducing PM 2.5 concentration levels in the Tokyo metropolitan area. Selecting 56 control measures, we consider the decision of whether each control measure should be installed. A detailed dataset on pollutant emissions is obtained from the JATOP [33]. The EPA [46] provides information on the abatement costs and elimination performances of the selected control measures. Using the integrated model and these data, we simulate two scenarios for meeting air quality standards and then compare the results. One scenario uses a cost-efficient strategy with the lowest total abatement cost. Another scenario ensures high equality among prefectures.

The remarkable findings of this paper are two-fold. First, if prefectures around Tokyo are going to meet air quality standards, they cannot do so by relying exclusively on their own efforts to reduce air pollutants (i.e., primary PM 2.5, NOx and SOx). This implies that it is necessary to coordinate with prefectures outside the Tokyo metropolitan area because of their large external contributions to PM 2.5 concentration levels around Tokyo. Second, total abatement costs are dramatically different between the two strategies. Based on the assumption that the concentration reduction from the external prefectures is 3.5 μg/m^3^, it will cost approximately 142.7 billion yen to meet the air quality standards using the cost-efficient strategy. In contrast, another strategy, which offers high equality among prefectures, costs approximately 416.3 billion yen; thus, the latter costs 2.9 times more than the former. This result implies that there is a large tradeoff between cost-efficiency and equality. Therefore, in order to ensure cleaner air, it is important that authorities make informed decisions when selecting a strategy.

We conclude this paper by acknowledging two limitations: First, we use pollutant emissions data from 2005, estimated by the JATOP [33] because of the lack of sufficient monitored data for Japan. Recently, the Japanese EPA has declared an intention to increase the number of ambient monitoring stations. Therefore, it is important that future studies update the scenario analysis with the latest monitoring data. Second, ammonia and volatile organic compounds, as secondary PM 2.5, are not considered in this paper because of the lack of available data on these pollutants. Moreover, aerosol researchers have not perfectly clarified the particle conversions of these pollutants, particularly VOCs, into PM 2.5 in the atmosphere [48]. These omitted pollutants must be incorporated into simulation models in the future.

## Supporting information

S1 Table List of fuel types. We cannot identify fuel types used in the electricity generation sector. Therefore, we treat all of them as one virtual fuel type.
(PDF)

S2 Table List of industries. (PDF)

S3 Table Reduction rates and emissions reductions by prefecture under the CES and URS scenarios. S3 Table presents the results under the CES and URS scenarios with the assumption of external concentration reductions of 5.0 μg/m^3^ instead of 3.0 μg/m^3^. This assumption enables every prefecture to easily meet the air quality standards. In the URS scenario, the uniform reduction rates are set at 30% in order to satisfy the air quality standard. On the other hand, under the CES scenario, the reduction rates also vary across prefectures and across pollutants, similar to those shown in Table 3. The emissions reductions decrease relative to those in Table 3 because the external reductions increase. In each prefecture, the total abatement costs are also smaller than those in Table 4. Under the CES scenario, the total cost is only approximately 1 billion yen, compared with 142.7 billion yen in Table 4. As shown in S4 Table, the gap in total costs between the CES and URS scenarios is extremely large at 30.3-fold, compared with 2.9-fold in Table 4, suggesting that the tradeoff between equality and cost-efficiency stands out as the external reductions increase.
(PDF)

S4 Table Total abatement cost by prefecture under the CES and URS scenarios. All units are 1 million yen, different from the units in Table 4. S3 Table presents the results under the CES and URS scenarios with the assumption of external concentration reductions of 5.0 μg/m^3^ instead of 3.0 μg/m^3^. This assumption enables every prefecture to easily meet the air quality standards. In the URS scenario, the uniform reduction rates are set at 30% in order to satisfy the air quality standard. On the other hand, under the CES scenario, the reduction rates also vary across prefectures and across pollutants, similar to those shown in Table 3. The emissions reductions decrease relative to those in Table 3 because the external reductions increase. In each prefecture, the total abatement costs are also smaller than those in Table 4. Under the CES scenario, the total cost is only approximately 1 billion yen, compared with 142.7 billion yen in Table 4. As shown in S4 Table, the gap in total costs between the CES and URS scenarios is extremely large at 30.3-fold, compared with 2.9-fold in Table 4, suggesting that the tradeoff between equality and cost-efficiency stands out as the external reductions increase.
(PDF)
