# Correction: Cost-efficient strategy for reducing PM 2.5 levels in the Tokyo metropolitan area: An integrated approach with air quality and economic models

**DOI:** 10.1371/journal.pone.0211255

**Published:** 2019-01-17

**Authors:** Yushi Kunugi, Toshi H. Arimura, Kazuyuki Iwata, Eiji Komatsu, Yoshie Hirayama

The images for Figs 1 and 2 are incorrectly switched. The image that appears as Fig 1 should be Fig 2, and the image that appears as Fig 2 should be Fig 1. The figure captions appear in the correct order.

**Fig 1:**
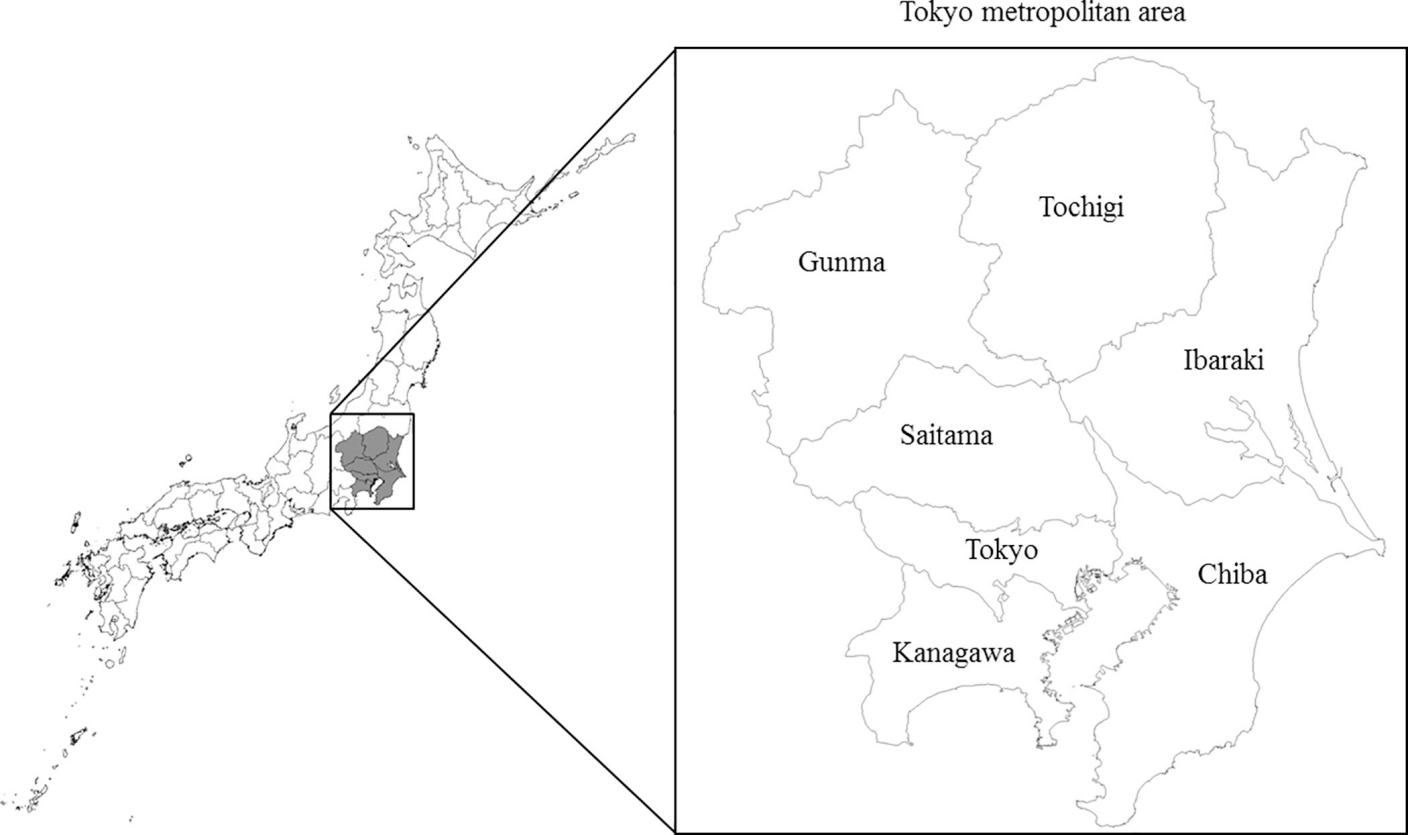
Seven prefectures in the Tokyo metropolitan area.

**Fig 2:**
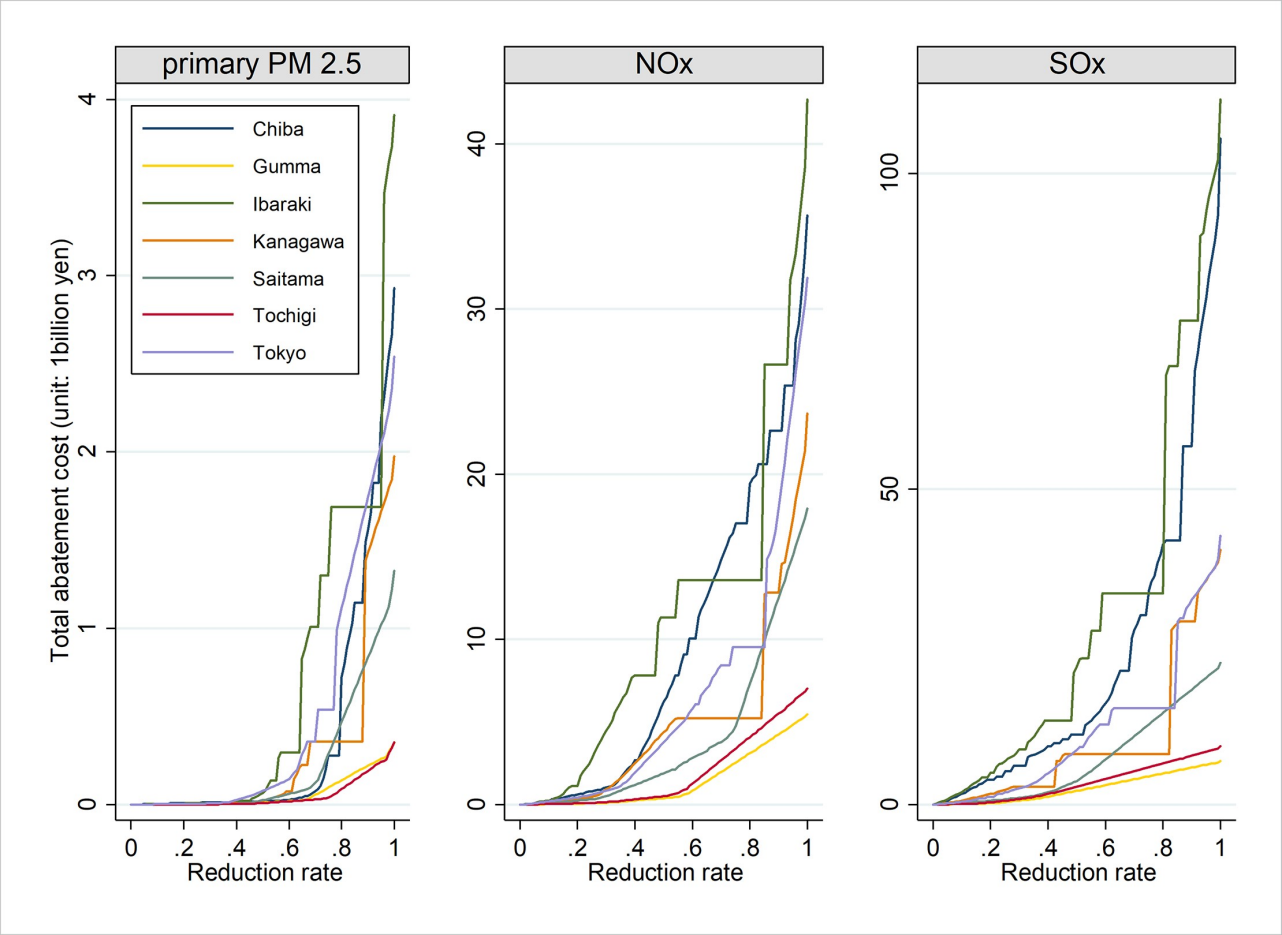
Relationship between total abatement costs and reduction rates by pollutant and by prefecture.
