# Supplementary material for: Cost-efficient strategy for reducing PM 2.5 levels in the Tokyo metropolitan area: An integrated approach with air quality and economic models
Source: PLoS One. 2018 Nov 26;13(11):e0207623. doi: 10.1371/journal.pone.0207623 (PMC6261045; doi:10.1371/journal.pone.0207623)
Supplement: S1 Table — We cannot identify fuel types used in the electricity generation sector. Therefore, we treat all of them as one virtual fuel type. (PDF) [file pone.0207623.s001.pdf]

Asphalt	Fuel oil	Natural gas
Black liquor recovery	Fuel oil A	Natural gas liquid
Blast furnace gas	Fuel oil C	No classification (industrial waste sector)
City gas	Gasoline	Other fuel oil, paraffin, etc.
Coal	Infectious waste	Petroleum coke
Coal coke	Jet fuel oil	Recycle oil
Coal tar	Kerosene	Refinery gas
Coke oven gas	Linz-Donawitz converter Gas	Refuse Derived Fuel (RDF)
Coking coal	Liquefied natural gas (LNG)	Steam coal
Crude oil	Liquefied petroleum gas (LPG)	Waste solid fuel / Refuse derived fuel (RDF)
Diesel oil	Lubricating oil	Wasted tire
Electric furnace gas	Mixed gas (MXG)	Wood
Electric generation sector	Naphtha	
