# Supplementary material for: Cost-efficient strategy for reducing PM 2.5 levels in the Tokyo metropolitan area: An integrated approach with air quality and economic models
Source: PLoS One. 2018 Nov 26;13(11):e0207623. doi: 10.1371/journal.pone.0207623 (PMC6261045; doi:10.1371/journal.pone.0207623)
Supplement: S2 Table — (PDF) [file pone.0207623.s002.pdf]

Electricity generation	Iron and steel
Foods	Nonferrous metals
Pulp and paper product	Business / Services
Chemicals	General waste
Petroleum and coal	Industrial solid waste / Industrial waste
Ceramics, stone and clay	
