# Supplementary material for: Cost-efficient strategy for reducing PM 2.5 levels in the Tokyo metropolitan area: An integrated approach with air quality and economic models
Source: PLoS One. 2018 Nov 26;13(11):e0207623. doi: 10.1371/journal.pone.0207623 (PMC6261045; doi:10.1371/journal.pone.0207623)
Supplement: S4 Table — All units are 1 million yen, different from the units in Table 4. S3 Table presents the results under the CES and URS scenarios with the assumption of external concentration reductions of 5.0 μg/m3 instead of 3.0 μg/m3. This assumption enables every prefecture to easily meet the air quality standards. In the URS scenario, the uniform reduction rates are set at 30% in order to satisfy the air quality standard. On the other hand, under the CES scenario, the reduction rates also vary across prefectures and across pollutants, similar to those shown in Table 3. The emissions reductions decrease relative to those in Table 3 because the external reductions increase. In each prefecture, the total abatement costs are also smaller than those in Table 4. Under the CES scenario, the total cost is only approximately 1 billion yen, compared with 142.7 billion yen in Table 4. As shown in S4 Table, the gap in total costs between the CES and URS scenarios is extremely large at 30.3-fold, compared with 2.9-fold in Table 4, suggesting that the tradeoff between equality and cost-efficiency stands out as the external reductions increase. (PDF) [file pone.0207623.s004.pdf]

Prefecture	CES scenario				URS scenario				Difference
	primary PM 2.5	NOx	SOx	Total (A)	primary PM 2.5	NOx	SOx	Total (B)	(B)/(A)
Chiba	75.0	172.6	0.0	247.6	11.7	1,015.9	6,192.8	7,220.4	29.2
Gumma	25.0	71.6	30.2	126.8	0.9	106.9	632.7	740.6	5.8
Ibaraki	23.0	0.0	0.0	23.0	8.6	4,466.5	8,724.4	13,199.5	574.3
Kanagawa	73.8	0.0	0.0	73.8	4.5	917.7	2,794.0	3,716.1	50.3
Saitama	286.8	154.5	0.0	441.3	3.4	521.5	1,064.6	1,589.6	3.6
Tochigi	1.8	0.0	0.0	1.8	1.1	152.0	905.1	1,058.2	580.5
Tokyo	108.6	0.0	0.0	108.6	6.3	848.7	2,575.4	3,430.4	31.6
Total	594.0	398.7	30.2	1,022.9	36.5	8,029.3	22,889.2	30,954.9	30.3
